# Cu, Zn-superoxide dismutase 1 (SOD1) is a novel target of Puromycin-sensitive aminopeptidase (PSA/NPEPPS): PSA/NPEPPS is a possible modifier of amyotrophic lateral sclerosis

**DOI:** 10.1186/1750-1326-6-29

**Published:** 2011-05-07

**Authors:** Guijie Ren, Zhongcai Ma, Maria Hui, Lili C Kudo, Koon-Sea Hui, Stanislav L Karsten

**Affiliations:** 1 Division of Neuroscience, Los Angeles Biomedical Research Institute at Harbor-UCLA Medical Center, Torrance, CA 90502, USA; 2 Department of Biochemistry and Molecular Biology, Medical College, Shandong University, Jinan, Shandong, 250012, China; 3 Nathan S. Kline Institute for Psychiatric Research, New York University School of Medicine, Orangeburg, NY 10962, USA; 4 NeuroInDx Inc., 1655 East 28th Street, Signal Hill, CA 90755, USA; 5 Department of Neurology, David Geffen School of Medicine at UCLA, Los Angeles, CA 90095, USA

## Abstract

Accumulation of misfolded neurotoxic Cu, Zn-superoxide dismutase-1 (SOD1) protein found in both familial and sporadic amyotrophic lateral sclerosis (ALS) is recognized as an important contributing factor of neuronal cell death. However, little is known about the mechanisms controlling the accumulation and turnover of SOD1 protein. Puromycin-sensitive aminopeptidase (PSA/NPEPPS) was recently identified as a major peptidase acting on neurotoxic TAU protein and protecting against TAU-induced neurodegeneration. In addition, recent report implicated PSA/NPEPPS in the direct removal of neurotoxic polyglutamine repeats. These combined data suggest that PSA/NPEPPS might represent a novel degradation pathway targeting pathologically aggregating neurotoxic protein substrates including SOD1. Here, we report that PSA/NPEPPS directly regulates SOD1 protein abundance and clearance via proteolysis. In addition, PSA/NPEPPS expression is significantly decreased in motor neurons of both *SOD*^*G93A *^transgenic mice and sporadic ALS patients, suggesting its possible contribution to the disease pathogenesis. These results implicate SOD1 as a new target protein of PSA/NPEPPS and point to the possible neuroprotective role of PSA/NPEPPS in ALS.

## Findings

Familial amyotrophic lateral sclerosis (FALS) represents about 10% of ALS cases. It is most frequently inherited as an autosomal dominant trait [1]. In about 20% of FALS patients, at least one type of mutation in Cu, Zn-superoxide dismutase-1 (SOD1) can be found [2]. The ubiquitous SOD1 protein converts superoxide radical anions to oxygen and hydrogen peroxide. More than a hundred SOD1 gain-of-function mutations have been identified, which may contribute to increased oxidative stress, altered copper metabolism, protein aggregation, excitotoxicity, or altered axonal transport [3]. Several other genes, including alsin, senataxin, vesicle-associated membrane protein-associated protein B, angiogenin, and TAR DNA binding protein, have also been found to be associated with ALS [4,5]. However, about 90% of ALS cases have no clear genetic cause, and the condition is referred to as sporadic amyotrophic lateral sclerosis (SALS) [4].

One of the hypotheses explaining selective motor neuron degeneration in ALS is the toxicity of intracellular protein aggregates. One of the major protein aggregates commonly found in FALS is the misfolded SOD1 protein [6-8]. Not surprisingly, aggregates of mutated SOD1 protein are found in neurons and astrocytes of SOD1 transgenic animals, including *G37R, G85R *and *G93A *models, and the degree of SOD1 accumulation strongly correlates with motor neuron dysfunction [9]. Immunohistochemical studies have also localized these inclusions predominantly to motor neurons, and in some cases, astrocytes [10]. Wild-type misfolded SOD1 is also found in the spinal cord extracts of SALS patients and may play a role in the etiology of SALS [6,11,12].

Accumulation of abnormally folded proteins and peptides is a key feature of many neurodegenerative diseases, including Alzheimer's disease, Huntington's disease and ALS [13-16]. The presence of misfolded SOD1 protein in both familial and sporadic ALS presents a credible explanation of motor neuron specific death in ALS [6,8,11,12,17-19]. Recent work convincingly demonstrated that wild-type SOD1 and mutant SOD1 share a conformational epitope prone to oxidation and therefore exert a strong neurotoxic effect [11,12,20]. These data strongly support the hypothesis that either mutated or wild-type SOD1 misfolding contributes to disease pathogenesis in both familial and sporadic ALS. Recent studies describing RNAi based allele-specific silencing of mutant SOD1 convincingly demonstrated that lowering the levels of mutant SOD1 protein produces a significant therapeutic benefit in SOD1^G93A ^mice [21]. This shows that targeting SOD1 is a viable and important therapeutic strategy for which identification and characterization of mechanisms controlling SOD1 protein degradation would play a central role. Unfortunately, the causes of SOD1 misfolding/accumulation, and more importantly, the mechanisms of the clearance of pathological aggregates remain unclear, which may very well be a valid target of novel therapeutic approaches for ALS.

Recently, we have identified puromycin-sensitive aminopeptidase (PSA, also known as NPEPPS) as a novel modifier of TAU-induced neurodegeneration with neuroprotective effects via direct proteolysis of TAU protein [22,23], which was later confirmed by others [24]. Another recent report implicated PSA as the major peptidase digesting polyglutamine sequences in Huntington's disease [25]. In addition, it was recently hypothesized that neuroprotective effect of PSA/NPEPPS may be linked to the autophagy system and several other neurotoxic targets *in vitro*, including polyQ-expanded huntingtin exon-1, ataxin-3, mutant α-synuclein, and SOD1 [26]. These results suggest that PSA/NPEPPS may represent a universal neuroprotective mechanism acting on pathologically aggregating neurotoxic proteins substrates, including SOD1. However, the questions whether PSA/NPEPPS is capable of removing SOD1 protein directly through its proteolytic activity and whether it contributes to the pathogenesis of familial and sporadic ALS remain unanswered.

Here, we investigated the role of PSA/NPEPPS in SOD1 protein clearance *in vitro *in cell culture and cell-free systems and evaluated the levels of PSA/NPEPPS in both human postmortem SALS motor neurons and murine ALS model tissues.

**Figure 1:**
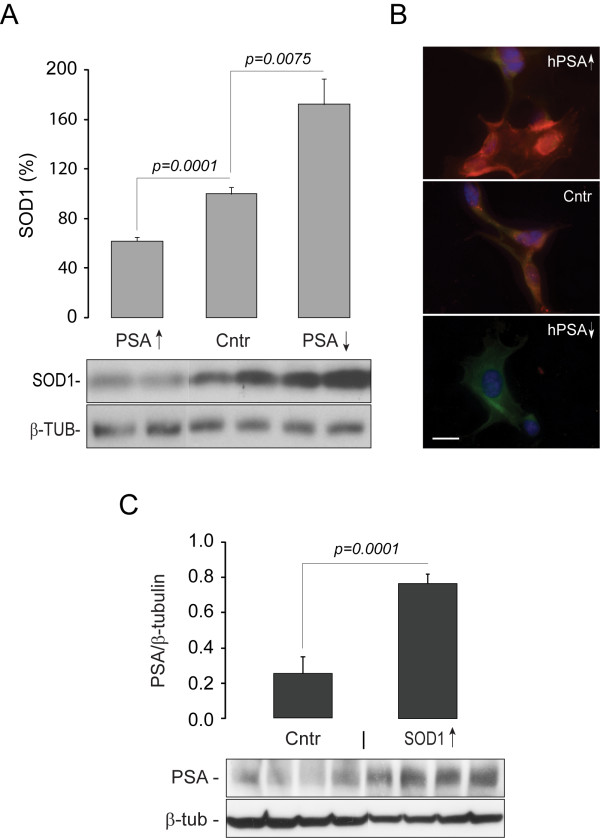
***In vitro *studies in neuroblastoma cell lines demonstrate co-regulation of PSA/NPEPPS and SOD1**. **A**. Western blot analysis shows decrease of endogenous SOD1 in response to PSA/NPEPPS overexpression (PSA↑) and accumulation of SOD1 in response to PSA/NPEPPS inhibition (PSA↓) with Stealth-RNAi in human SH-SY5Y neuroblastoma cell line. **B**. Representative immunocytochemistry demonstrates a notable decrease (top) and a rapid accumulation (bottom) of SOD1 protein (green) in response to PSA/NPEPPS overexpression (PSA↑; top; red) and PSA/NPEPPS inhibition (PSA↓; bottom), respectively, in SH-SY5Y cells. The middle image represents normal control. PSA/NPEPPS is red, SOD1 is green, and the nucleus is blue as stained with DAPI nuclear marker. Scale bar 20 μm. **C**. PSA/NPEPPS expression demonstrated by Western blot is up-regulated in response to SOD1 overexpression in mouse N1E-115 neuroblastoma cell line. Because mouse SOD1 overexpression construct was used, we utilized mouse neuroblastoma cell line to avoid possible side effects of heterologous expression of a mouse protein in a human cell line. For each comparison, three independent experiments were performed (n = 3) with at least three independent replicates in each experiment. Representative Western blot and immunocytochemistry images are shown. Error bars represent standard deviations.

We first tested whether PSA/NPEPPS overexpression is able to reduce the abundance of endogenous SOD1 in human neuroblastoma SH-SY5Y cells. SH-SY5Y cells were transfected with human PSA/NPEPPS overexpression vectors, pCMV6-XL-hPSA and then the endogenous SOD1 was analyzed with Western blot and immunocytochemistry according to standard protocols and previously published protocols, respectively [27]. The highest transfection peaks for PSA/NPEPPS overexpression was noticed at 48 and 72 hours (data not shown). While the analysis of cells at 24 and 48 hours after transfection did not show any noticeable changes in SOD1 protein expression (data not shown), cells incubated for 72 hours post-transfection demonstrated a significant decrease (nearly 50% reduction; *p *= 0.0001) of endogenous SOD1 protein (Figure 1A and 1B). We also investigated the effect of knocking down PSA/NPEPPS expression using RNA interference (Stealth-RNAi) on SOD1 levels in SH-SY5Y cells. Reduction of PSA/NPEPPS expression resulted in an accumulation (~2-fold; *p *= 0.0075) of endogenous SOD1 protein within 72 hours (Figure 1A, B). Our above *in vitro *experiments in human neuroblastoma cell line support the recent observation that PSA/NPEPPS is a modulator of SOD1 protein abundance in 293A cells [26].

Moreover, we also studied whether overexpression of SOD1 affects the expression of endogenous PSA/NPEPPS. In mouse neuroblastoma cell line N1E-115 transfected with mouse SOD1 overexpression vectors pCMV6-XL-SOD1, mouse PSA/NPEPPS protein expression was upregulated up to 4-fold (*p *= 0.0001) (Figure 1C). Thus, it seems that PSA/NPEPPS expression is directly proportional to the levels of SOD1 protein and the elevation of SOD1 leads to significant increase in PSA/NPEPPS protein expression. These observations taken together suggest that PSA/NPEPPS is indeed a direct endogenous regulator of SOD1 protein abundance and its expression is controlled in response to alterations of intracellular SOD1 levels through a positive feed-back mechanism. A similar regulation of PSA expression has been reported in PC12 cells that overexpress hungingtin exon 1 containing polyQ sequences, in which PSA/NPEPPS expression is highly induced [28]. These results suggest that there may be a feed-back self-protective mechanism in cells by which PSA/NPEPPS expression is regulated in response to the abundance of aggregated toxic proteins. This further implies the important physiological neuroprotective role of PSA/NPEPPS.

Using human neuroblastoma SH-SY5Y cells we demonstrated a strong linkage of PSA/NPEPPS to SOD1 accumulation/clearance (Figure 1A and 1B). This effect may be mediated by direct interaction of PSA/NPEPPS with SOD1 resulting in SOD1 protein digestion similar to PSA/NPEPPS interaction with TAU-protein [22,23]. However, it is plausible that PSA/NPEPPS has no direct interaction with SOD1, and acts via cleavage of another intermediary, such as the autophagy system [26] or ptoteosome system [25]. Our recent studies of hPSA transgenic mice overexpressing human PSA/NPEPPS at nearly 3-fold revealed that it has no significant effect on either autophagy or proteosome degradation system [29]. Nevertheless, to test the hypothesis that human PSA/NPEPPS can act directly on human SOD1 two types of experiments were performed in the cell-free system using purified SOD1 and PSA/NPEPPS proteins.

**Figure 2:**
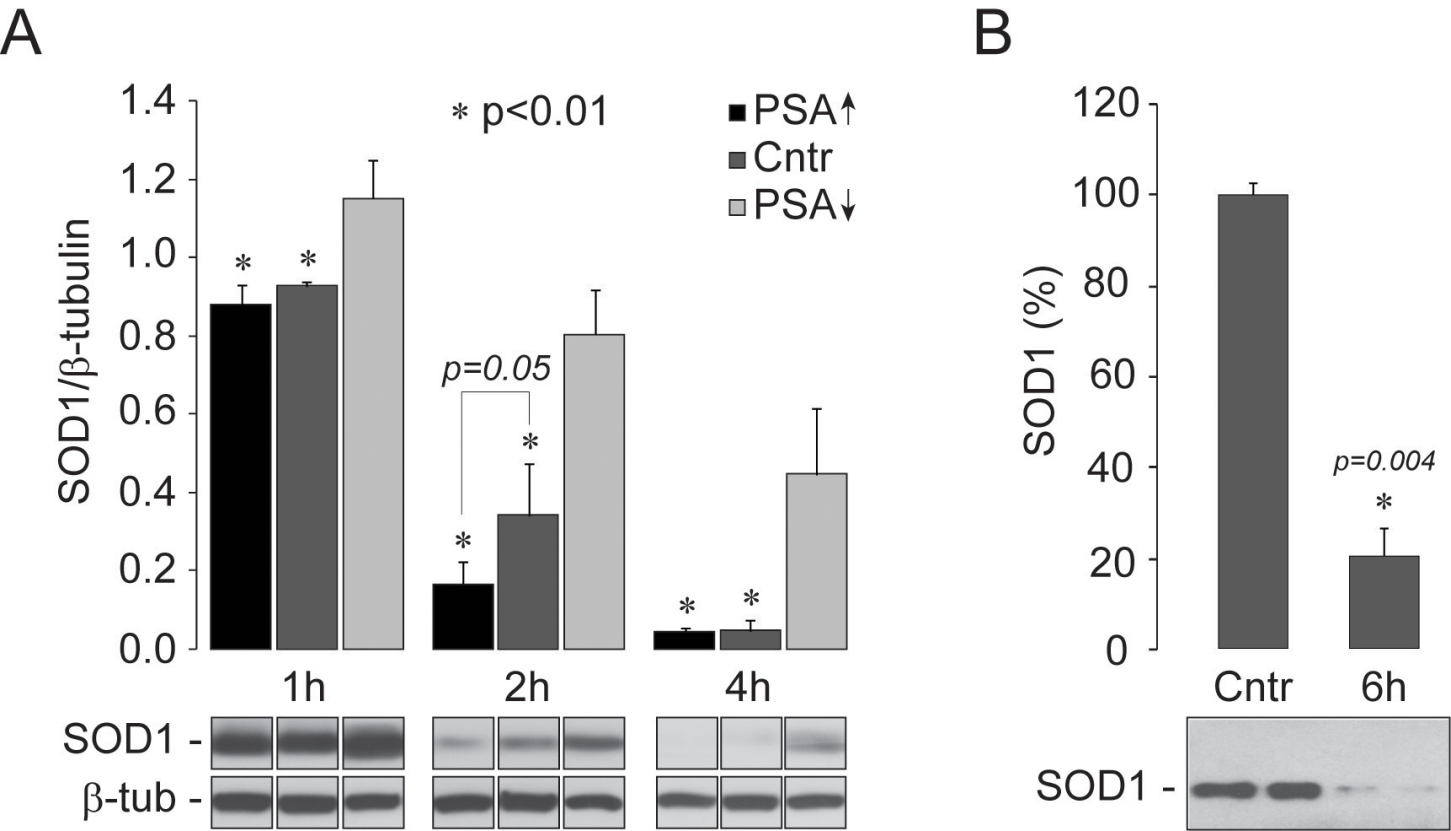
**Experiments in the cell-free system by Western blot analysis confirm direct interaction of PSA/NPEPPS and SOD1**. **A**. Purified human SOD1 was incubated with post-microsomal protein fractions of SH-SY5Y cells transfected with PSA/NPEPPS overexpressing vector (PSA↑), blank vector (Cntr) or Stealth-RNAi specific for hPSA/NPEPPS (PSA↓); and the exhibited SOD1 levels are reversely proportional to the levels of PSA/NPEPPS protein. Independent experimental triplicates are shown. **B**. Incubation of purified human SOD1 with purified rat PSA/NPEPPS for 6 hours demonstrates up to 80% proteolysis of human SOD1. Controls contained human SOD1 only. The gel bands in **B **demonstrate the results from two independent co-incubation experiments. Only representative images are shown. Error bars represent standard deviations.

First, we prepared post-microsomal protein extracts (S_3_) from cultured SH-SY5Y cells transfected with PSA/NPEPPS overexpression vectors, PSA/NPEPPS-specific RNAi, and corresponding controls, as described previously [30]. Co-incubation of post-microsomal fractions with purified human SOD1 was carried out at 37°C for one, two and four hours in the Bicine buffer, pH 7.0 with 0.2 mM DTT. One microgram of SOD1 highly purified from human erythrocytes (Sigma-Aldrich, MO) was co-incubated with 25 μg of S_3 _protein extracts isolated from nontransfected cells (control), cells transfected with PSA/NPEPPS overexpression vectors (PSA↑), and cells transfected with Stealth-RNAi (Invitrogen, CA) specific for human PSA/NPEPPS (PSA↓). The reactions were terminated by the addition of Laemmli SDS sample buffer and boiling for 5 min. Subsequently SOD1 proteins that remained in the reaction mixtures were analyzed by Western blot with SOD1 antibodies (MBL Intl, MO). While one hour incubation did not produce any noticeable effect on SOD1, two hours incubation resulted in a significantly increased SOD1 degradation rate (2-fold, *p *= 0.04) with the post-microsomal extracts containing elevated PSA/NPEPPS compared to using the post-microsomal extracts from control cells. Prolonged incubation for up to 4 hours resulted in the degradation of SOD1 in both experimental and control samples due to the presence of other proteases in the post-microsomal protein extracts. On the contrary, with post-microsomal protein extracts from PSA/NPEPPS-RNAi treated cells with knocked down PSA/NPEPPS, the rates of SOD1 degradation were significantly reduced (*p *< 0.01) at all time points in comparison to using protein extracts with elevated PSA/NPEPPS and controls (Figure 2A). The decrease in SOD1 degradation rate ranged from 1.24-fold (*p *= 0.04) after 1 hour to 9.7-fold (*p *< 0.01) after 4 hours of co-incubation. Experimental data presented above using the purified SOD1 and post-microsomal protein fractions that lack the autophagy system activity indicate that without the involvement of autophagy system PSA/NPEPPS is still capable of hydrolyzing SOD1 independently.

Although post-microsomal protein fractions are free of autophagosomal vesicles, other cytoplasmic peptidases may contribute to SOD1 degradation process [31]. To further confirm that PSA/NPEPPS indeed acts on SOD1 protein directly and is capable of degrading it, we incubated purified human SOD1 with well-purified PSA/NPEPPS in a cell-free system. PSA/NPEPPS was purified from brains of adult male Sprague-Dawley rats (250-300 g) and its activity was measured with 20 μM Leu βNA (Leu-naphthylamide) as described previously [32]. For PSA-SOD1 incubation experiments, digestion of highly purified human SOD1 (Sigma-Aldrich, MO) was carried out at 37°C for 6 hrs in 50 mM Bicine buffer, pH 7.0, containing 0.2 mM DTT, at a molar ratio of 1:6 (PSA:SOD1). The reaction was terminated by 5% perchloroacetic acid and analyzed by Western blot as described above. After 6 hours of incubation, full-length SOD1 was greatly diminished of its original amount (about 80% decrease, *p *= 0.004) (Figure 2B). These results further strengthen the hypothesis of PSA/NPEPPS-specific SOD1 degradation through its direct proteolytic activity similar to the effect of PSA/NPEPPS on TAU-protein [22,23]. Moreover, our studies with post-microsomal protein fractions showed that such protein fractions even from cells that lack PSA/NPEPPS expression are also capable of digesting SOD1 protein at a low rate, which suggests that there may be other unknown PSA-independent mechanisms responsible for SOD1 hydrolysis, which remain to be verified in the future. Based on observations made by us and by others [26], PSA/NPEPPS may regulate SOD1 degradation through both direct (i.e. its proteolytic activity) and indirect (such as autophagy system involved) mechanisms. Our findings indicate that SOD1 protein may be removed directly through the increased activity of PSA/NPEPPS, and implicate this enzyme as an important modulator of SOD1-induced motor neuron degeneration in ALS.

**Figure 3:**
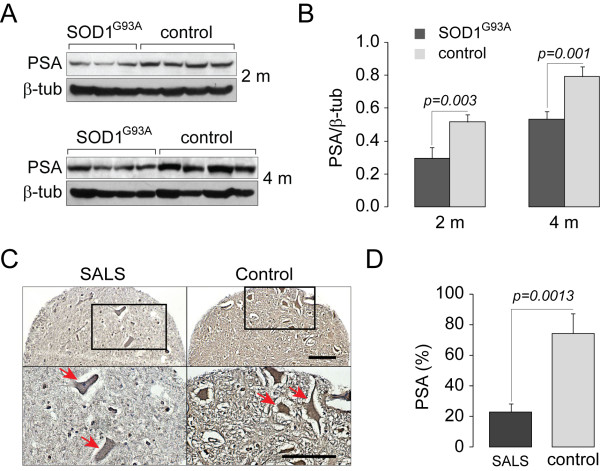
**PSA/NPEPPS is significantly decreased in both SOD1**^**G93A **^**transgenic mice and motor neurons of postmortem SALS patients**. **A, B**. PSA/NPEPPS protein expression analyzed with Western blot is significantly decreased in the spinal cord of 2 months old (2 m) adult presymptomatic (n = 4) and 4 months old (4 m) symptomatic (n = 4) SOD1^G93A ^transgenic mice compared to the corresponding littermate 2 (n = 4) and 4 (n = 4) months old wild-type mice. **C, D**. A high-throughput immunohistochemical analysis using SALS tissue microarray reveals a significant decrease of PSA/NPEPPS-positive anterior horn motor neurons in SALS patients (n = 19) compared to healthy controls (n = 6). **C**. Representative spinal cord anterior horn tissue cores from SALS and control subjects show that PSA/NPEPPS protein expression is reduced in SALS motor neurons. Scale bar 20 μm. **D**. Bar graphs summarizing the findings from SALS tissue microarray show significant decrease of PSA/NPEPPS in SALS motor neurons. On average, each SALS (n = 19) and control (n = 6) subject was represented by ten spinal cord anterior horn tissue cores [38]. Error bars represent standard deviations.

Even though it seems that SOD1 overexpression *in vitro *may trigger PSA/NPEPPS upregulation (Figure 1C), the response of PSA/NPEPPS to the expression of mutated and dysfunctional SOD1 found in FALS is unknown. To investigate the effect of long term constitutive overexpression of mutated SOD1 *in vivo*, we first studied the expression levels of PSA/NPEPPS in *SOD1*^*G93A *^transgenic mice and control littermates [33]. *SOD1*^*G93A *^mice, strain *B6SJL-Tg (SOD1-G93A)1Gur*, were obtained from The Jackson Laboratory (Bar Harbor, ME). This strain expresses the transgene under the control of the endogenous human promoter [34]. Female *SOD1*^*G93A *^transgenic and non-transgenic control littermates were sacrificed at the age before the onset of the known disease pathology (2 months) and during the symptomatic stage (4 months). Western blot analysis using antibodies against PSA/NPEPPS was performed with the protein lysates extracted from the spinal cords of each group of animals. All animal protocols were in accordance with the NIH Guide for the Care and Use of Laboratory Animals and were approved by the Los Angeles Biomedical Research Institute and Nathan S. Kline Institute for Psychiatric Research animal studies committees. These experiments demonstrated significant reduction of PSA/NPEPPS in the tissues of *SOD1*^*G93A *^transgenic mice at both ages tested (*p *= 0.003 and *p *= 0.001 respectively) compared to control mice, suggesting that contrary to the short-term effect *in vitro *prolonged accumulation of mutated SOD1 negatively regulates PSA/NPEPPS expression *in vivo *(Figure 3A and 3B).

Although the presence of misfolded SOD1 in the motor neurons of sporadic ALS patients is still a debated topic evidenced by several contradicting reports [6,8,11,12,17-19,35,36], recently published data points to the importance of SOD1 in the pathogenesis of the sporadic disease [11]. It was demonstrated that oxidized wild-type SOD1 shares similar structural and neurotoxic features with mutated SOD1. It appears that SOD1 oxidation process leads to specific conformational changes in SOD1 creating an epitope identical to mutated and neurotoxic SOD1 [11]. In over 50% of SALS patients such aberrant wild-type SOD1 are detected, and SOD1 extracted from tissue samples of SALS patients, but not SOD1 from healthy controls, inhibited axonal transport, pointing to its strong neurodegenerative potential [11]. These results suggest that abnormal conformations of oxidized wild-type SOD1 could indeed mediate motor neuron toxicity in SALS similar to the role of mutated SOD1 in FALS [37].

We then studied whether PSA/NPEPPS expression was altered in SALS patients. Post-mortem paraffin-embedded clinically relevant brain and spinal cord tissue blocks from SALS patients (n = 19) and normal controls (n = 6) were provided by the UCLA Department of Pathology and the National Neurological AIDS BANK (NNAB), and used for tissue microarray (TMA) construction at the UCLA Tissue Array core facility (http://www.genetics.ucla.edu/tissuearray) as described previously [38]. Estimation of PSA/NPEPPS expression was performed in spinal cord anterior horn motor neurons of SALS and control subjects. Briefly, for the estimation of relative PSA/NPEPPS expression, the total number of PSA/NPEPPS positive motor neurons was divided by the total number of NeuN positive motor neurons to derive the PSA expression index (PEI) for a given tissue core. Then, the average PEI for each tissue type and individual was calculated and used to derive the mean PEI for SALS and control subjects. The significance was estimated based on a Student's *t*-test (*P *< 0.01). The following primary antibodies were used: mouse monoclonal anti-neuronal nuclei (NeuN; 1:100; Chemicon) and goat polyclonal anti-PSA (1:1000, Millipore). This high-throughput immunohistochemical analysis of TMA showed a significant decrease of PSA/NPEPPS protein expression in the SALS motor neurons (*p *= 0.0013, Figure 3C and 3D). For the first time, we demonstrated that decreased expression of PSA/NPEPPS may be a novel contributory factor to the pathogenesis of ALS, which leads to the impaired clearance of accumulated SOD1.

Our *in vitro *results with murine neuroblastoma cell line overexpressing SOD1 demonstrated an increase in PSA/NPEPPS protein expression (Figure 1C). However, the evidence *in vivo *show that the levels of PSA/NPEPPS protein are dramatically decreased in the transgenic mice overexpressing mutated (*G93A*) form of human SOD1 and in postmortem spinal cord tissues, more specifically motor neurons, of SALS patients (Figure 3). Two possible mechanisms may explain these conflicting observations *in vitro *and *in vivo*: 1. the rapid upregulation of PSA/NPEPPS is just the early-response to elevated intracellular SOD1; however, the prolonged and constitutive elevation of SOD1 eventually leads to the decreased expression of PSA/NPEPPS; 2. In SALS disease, the physiological feed-back regulatory mechanism of PSA/NPEPPS expression in response to SOD1 levels is impaired. The latter may represent a novel angle of view of ALS pathogenesis. Future studies of the interaction of PSA/NPEPPS with SOD1 would be prerequisite to understand its biological mechanisms. In particular, the cross between recently developed hPSA transgenic mice [29] and *SOD1*^*G93A *^mice [33] with subsequent analysis of double transgenic progeny in respect to *SOD1*^*G93A *^protein accumulation would be valuable. Further cell free co-incubation experiments using mutated forms of SOD1 would provide additional information on the specificity of interaction with PSA/NPEPPS. These and other experiments would shed new light on the role of PSA/NPEPPS in the pathogenesis of ALS and may provide novel therapeutic and diagnostic approaches for ALS.

In summary, we present the first evidence that PSA/NPEPPS is a major protease to directly digest SOD1, which may be analogous to its role in TAU-induced neurodegeneration [22-24]. More importantly, its expression is attenuated in both murine ALS model and SALS patients, which suggests its potential contribution to ALS pathogenesis. However, additional functional and etiological studies are needed to fully evaluate the role of PSA/NPEPPS in ALS and its possible use as a therapeutic target facilitating SOD1 protein clearance.

## Abbreviations

ALS: amyotrophic lateral sclerosis; FALS: familial amyotrophic lateral sclerosis; PSA/NPEPPS: puromycin-sensitive aminopeptidase; SALS: sporadic amyotrophic lateral sclerosis; SOD1: Cu, Zn-superoxide dismutase-1

## Competing interests

The authors declare that they have no competing interests.

## Authors' contributions

GR, LP, MH carried out all the experiments. ZM maintained the transgenic mice colony, interpreted the data and drafted the manuscript. LCK, KSH, SLK designed the study, performed statistical analysis and drafted the manuscript.

All authors have read and approved the final manuscript.
